# Association between self-reported physical activity and incident atrial fibrillation in a young Korean population

**DOI:** 10.1038/s41598-019-40744-x

**Published:** 2019-03-12

**Authors:** Sung Ho Lee, Seungho Ryu, Jong-Young Lee, Dae Chul Seo, Byung Jin Kim, Ki-Chul Sung

**Affiliations:** 1 Division of Cardiology, Department of Internal Medicine, Kangbuk Samsung Hospital, Sungkyunkwan University School of Medicine, Seoul, Republic of Korea; 2 Department of Occupational and Environmental Medicine, Kangbuk Samsung Hospital, Sungkyunkwan University School of Medicine, Seoul, Republic of Korea; 3 Center for Cohort Studies, Total Healthcare Center, Kangbuk Samsung Hospital, Sungkyunkwan University School of Medicine, Seoul, Republic of Korea; 4 Department of Clinical Research Design & Evaluation, SAIHST, Sungkyunkwan University, Seoul, Republic of Korea

**Keywords:** Biophysics, Cardiovascular diseases, Atrial fibrillation

## Abstract

The association between physical activity (PA) and atrial fibrillation (AF) remains unclear. We thus investigated association of PA with the development of AF. Type and duration data for PA were acquired from 211,992 AF-free individuals using the validated Korean version of the International Physical Activity Questionnaire Short From between March 2002 and December 2014. Individuals were divided into four groups according to self-reported PA level and previous international PA guidelines: no PA with a sedentary lifestyle, insufficient PA, sufficient PA, and health-enhancing PA. AF was diagnosed by annual 12-lead electrocardiogram. During a median follow-up of 5.6 years, AF occurred in 304 participants (annual AF incidence rate 2.5/10,000 person-years). After adjusting for age and sex, PA level was not associated with AF incidence (P for trend = 0.26). The multivariable-adjusted hazard ratios (95% confidence interval) for incidental AF was 1.00 (0.74–1.37) for the insufficient PA group, 1.34 (0.91–1.97) for the sufficient PA group, and 1.27 (0.72–2.23) for the health-enhancing PA group compared with the no PA group (P for trend = 0.18). Thus, our study does not support an association between the risk of AF and PA level in a young Korean population.

## Introduction

Atrial fibrillation (AF) is the most commonly identified arrhythmia. It can lead to significantly increased morbidity and mortality^1^. Traditional risk factors for AF include advanced age, hypertension, diabetes mellitus, structural heart disease, alcohol abuse, pulmonary disease, and thyroid disease^2^. Vigorous long-term physical activity (PA) is reported to increase AF risk^3–6^, but controversy remains regarding the association between PA and AF. Previous studies found that long-term participation in endurance sports or rigorous PA increased the risk of AF in young and middle-aged men^3,7–9^. However, other studies reported that leisure-time PA at an older age was associated with decreased AF risk in the general population^8^. A prospective study of older men and women observed a reduction in AF risk with moderate-intensity PA but found no increase in risk with high-intensity PA^10^. These divergent results might be caused by the use of different definitions of PA or study population characteristics (e.g., age and sex). Most research about PA and risk of AF has focused on Caucasians, and studies on the relationship PA level and AF risk in Asians are scarce. Thus, we examined the influence of PA on the risk of developing AF in a large, general Korean population.

## Methods

### Study population

The Kangbuk Samsung Health Study is a cohort study of South Korean men and women 18 years of age or older who underwent a comprehensive annual health examination at Kangbuk Samsung Hospital Total Health Centers in Seoul or Suwon, South Korea^11^. More than 80% of participants were employees or spouses of employees of local companies or governmental organizations. In South Korea, the Industrial Safety and Health law requires annual or biennial health screening examinations of all employees. The remaining 20% of the participants voluntarily took part in the screening examinations. The study population was 234,170 individuals who participated in an annual comprehensive health screening examination at Kangbuk Samsung Hospital, Seoul, Korea, between March 2002 and December 2014. We excluded participants for whom data on smoking, alcohol intake, PA (n = 19,533), cancer history (n = 2,475), or baseline AF (n = 170) were missing. After these exclusions, 211,992 participants were eligible for analysis. The Institutional Review Board of Kangbuk Samsung Hospital approved this study. The need for informed consent was waived as we used only anonymized retrospective data routinely collected during the health screening process.

### Measurements

All individuals completed self-administered questionnaires related to their medical, social, smoking, and alcohol consumption history. Individuals were asked about their duration of education (high school graduate or less, community college or university graduate), smoking history (never, former, or current) and alcohol consumption (moderate <20 g/day or high ≥20 g/day). Blood pressure (BP) was measured in a sitting position with the arm supported at heart level. Hypertension (HTN) was defined as systolic BP ≥ 140 mmHg, diastolic BP ≥ 90 mmHg or self-reported history of HTN^12^. Diabetes mellitus (DM) was defined as a fasting serum glucose level ≥126 mg/dl or HbA1c ≥ 6.5%, self-reported history of diabetes, or current use of diabetes medication^13^. Body mass index (BMI) was calculated as weight in kilograms divided by height in meters squared. Obesity was defined according to recommendations for Asian populations^14^ with a BMI threshold for obesity ≥25 kg/m^2^. Abdominal ultrasonography was performed by experienced clinical radiologists, and fatty liver was diagnosed based on standard criteria^15^. Cardiovascular disease (CVD) was defined as a group of disorders of the heart and blood vessels, including coronary heart disease and stroke.

Laboratory measurements of serum biochemical parameters, including total cholesterol, triglycerides, high-density lipoprotein cholesterol (HDL-C), low-density lipoprotein cholesterol (LDL-C), glucose, insulin, insulin resistance, A1C, and high-sensitivity C reactive protein (hs-CRP) were the same as detailed elsewhere^11^.

### Assessment of physical activity

PA levels were assessed using the validated Korean version of the International Physical Activity Questionnaire Short Form (IPAQ-SF)^16^. From 2011 on, the validated Korean version of the IPAQ-SF was used to assess the weekly frequency of moderate- or high-intensity PA. Although IPAQ-SF had not been officially introduced, similar questionnaires were used before 2011. The IPAQ-SF measures the frequency and duration of moderate-to-high-intensity PA for more than 10 continuous minutes across all usual activities at work and home or during leisure for middle-aged individuals during a seven-day period^17^. Specific questions asked to gauge the number of times per week a participant engaged in PA were: “During the past 7 days, how many days did you undertake high-intensity PA, such as heavy lifting, aerobics, or fast bicycling?” or moderate-intensity PA e.g., carrying light loads, bicycling at a steady pace, or doubles tennis?” If the answer was “1 or more”, additional questions were asked, such as “How much time did you usually spend performing moderate- or high-intensity PA on one of those days^17^?” The metabolic equivalents (MET) level-minutes per week were calculated by assigning standardized MET values of 4 and 8 for moderate- and high-intensity activities, respectively^17^. Previous international guidelines recommended at least 150 minutes per week of moderate-intensity PA and 75 minutes per week of high-intensity PA^18,19^. Participants were divided into four groups according to IPAQ-SF and the above-mentioned guidelines: no PA with a sedentary lifestyle, insufficient PA^18,19^, sufficient PA^18,19^ and strenuous health-enhancing PA^17,19,20^. Participants were categorized into the insufficient PA group if they neither meet sufficient PA nor strenuous health-enhancing PA criteria. Participants in the sufficient PA group met any of the following criteria: (1) 3 or more days of high-intensity activity of at least 20 minutes per day, (2) 5 or more days of moderate-intensity activity at least 30 minutes per day, or (3) 5 or more days of any combination of moderate- or high-intensity activities achieving at least 600 MET-minutes/week^17^. Participants were categorized into the health-enhancing PA group if they met either of 2 criteria: (1) high-intensity activity on at least 3 days per week accumulating at least 1500 MET-minutes/week or (2) 7 days of any combination of moderate- and high-intensity activities achieving at least 3000 MET-minutes/week^17^.

### Follow-up and AF detection

Diagnosis of AF was based on annual 12-lead electrocardiography and was confirmed by an experienced cardiologist.

### Statistical analysis

Continuous variables were expressed as mean ± standard deviation (SD) for normally distributed variables or as median and interquartile range (IQR) if not normally distributed. Comparisons between groups were performed with one-way analysis of variance (ANOVA) with Tukey’s multiple comparison analysis for continuous variables. Categorical variables were expressed as percentages and compared between groups using the Chi-square test. We conducted a Cox proportional hazard regression to estimate the hazard ratios with 95% confidence interval (CI) for AF incidence according to PA level. The group that performed no PA with a sedentary life-style was used as the reference. We adjusted analyses for three different models. Model 1 was adjusted for age, sex, center (Seoul, Suwon), year of screening examination, smoking status (never, former, or current), alcohol intake (moderate <20 g/day or high ≥20 g/day), and education level (high school graduate or less, community college, or university graduate). Model 2 was adjusted for model 1 covariates and BMI, history of HTN, DM, and CVD. Model 3 was adjusted for the covariates of model 2 and hs-CRP. Statistical analysis of data was performed using PASW version 18 (SPSS, Chicago, IL, USA). Statistical tests were two-tailed, and p < 0.05 was considered statistically significant.

## Results

During a median follow-up of 5.6 years (IQR 2.7–8.4), 304 (0.14%) AF cases (278 men and 26 women) were diagnosed, corresponding to 2.5 cases of AF per 10,000 person-years. The mean age of participants with AF was 44.7 ± 10.4 years. Baseline characteristics of the study population according to the incidence of AF are presented in Table 1. Participants with incidental AF were more likely to be men of older age, current smokers and alcohol drinkers. They were more likely to have higher BMI, higher systolic and diastolic blood pressure, higher blood glucose, worse lipid profile, and more underlying obesity, fatty liver, HTN, and CVD than those with no AF. Table 2 shows baseline characteristics of participants according to PA level. The health-enhancing PA group was older and had fewer cardiovascular risk factors, including lower BMI, LDL-C, triglycerides, and blood insulin levels, higher HDL-C, a lower prevalence of obesity and fatty liver, and less alcohol intake than those with insufficient PA. A slightly higher proportion of people with DM, HTN, and existing CVD were included in the health-enhancing PA group compared to the no PA with a sedentary lifestyle group. Table 3 shows risk of incidental AF according to PA level. After adjusting for age and sex, PA level was not associated with AF incidence (P for trend = 0.26). After further adjustment for center, year of screening examination, smoking status, alcohol intake, education level, BMI, diabetes, HTN, CVD, and hs-CRP, PA level was not associated with AF incidence (model 3). The hazard ratio for incidental AF was 1.00 (95% CI 0.74–1.37) for the insufficient PA group, 1.34 (95% CI 0.91–1.97) for the sufficient PA group, and 1.27 (95% CI 0.72–2.23) for the health-enhancing group compared with the no PA group (P for trend = 0.18).

**Table 1:** Baseline characteristics of study participants according to incident AF.

Baseline characteristics	No AF (n = 211,688)	AF (n = 304)	p value
Gender, male (%)	129,765 (61.3)	278 (91.5)	<0.001
Age (years)^a^	37.7 (7.8)	44.7 (10.4)	<0.001
Height (cm)	167.7 (8.2)	171.5 (7.3)	<0.001
BMI (kg/m^2^)^a^	23.4 (3.1)	25.4 (3.0)	<0.001
Systolic BP (mm Hg)^a^	113.6 (13.6)	120.6 (15.2)	<0.001
Diastolic BP (mm Hg)^a^	73.4 (10.0)	78.9 (11.0)	<0.001
Higher education (%)^*^	164,058 (77.5)	218 (71.8)	0.041
Current smoker (%)	6,054 (28.6)	117 (38.5)	<0.001
Alcohol intake ≥20 g/day (%)	32,811(15.5)	84 (27.6)	<0.001
Fatty liver (%)	56,097 (26.5)	111 (36.8)	<0.001
Obesity (%)	61,812 (29.2)	167 (54.9)	<0.001
Diabetes mellitus (%)	5,715 (2.7)	9 (3.0)	0.802
Hypertension (%)	29,001 (13.7)	97 (31.9)	<0.001
Cardiovascular disease (%)	6,985 (3.3)	18 (5.9)	0.010
Insulin (µIU/mL)^b^	4.8 (3.3–6.9)	3.6 (5.4–8.1)	0.344
Glucose (mg/dL)^a^	94.0 (14.7)	96.0 (12.3)	0.018
Total cholesterol (mg/dL)^a^	194.3 (34.6)	200.6 (33.0)	0.001
LDL-C (mg/dL)^a^	113.7 (30.0)	116.7 (27.6)	0.083
HDL-C (mg/dL)^a^	55.6 (12.8)	52.0 (11.2)	<0.001
Triglycerides (mg/dL)^b^	102 (72–151)	130.5 (94.5–187)	<0.001
HOMA IR^b^	1.69 (1.22–2.28)	1.85 (1.37–2.47)	<0.001
hs-CRP (mg/L)^b^	0.04 (0.02–0.1)	0.06 (0.03–0.14)	<0.001

Data are presented as ^a^mean (standard deviation), ^b^median (interquartile range), or percentage.

AF, atrial fibrillation; BMI, body mass index; BP, blood pressure; LDL-C, low-density lipoprotein cholesterol; HDL-C, high-density lipoprotein cholesterol; HOMA IR, homeostasis model assessment of insulin resistance, hs-CRP, high sensitivity C-reactive protein. Cardiovascular disease was defined as coronary heart disease or stroke.

^*^≥college graduate.

**Table 2:** Baseline characteristics of study participants according to physical activity level.

Characteristics	Overall (n = 211,992)	Physical activity group				P for trend
		Sedentary (n = 117,571)	Insufficient (n = 61,869)	Sufficient (n = 23,309)	Health enhancing (n = 9,243)	
Age (years)^a^	37.8 (7.8)	36.8 (7.4)	38.3 (7.7)	39.8 (8.4)	40.8 (9.9)	<0.001
Gender, male (%)	129,951 (61.3)	63,136 (53.7)	46,278 (74.8)	15,104 (64.8)	5,370 (58.1)	<0.001
Height (cm)	167.7 (8.2)	166.8 (8.3)	169.4 (7.8)	167.9 (8.2)	166.8 (8.5)	<0.001
BMI (kg/m^2^)^a^	23.4 (3.1)	23.0 (3.2)	23.9 (3.0)	23.9 (2.9)	23.7 (2.9)	<0.001
Systolic BP (mmHg)^a^	113.6 (13.7)	112.3 (12.4)	115.2 (13.6)	115.3 (13.9)	115.7 (14.3)	<0.001
Diastolic BP (mmHg)^a^	73.4 (10.0)	72.4 (9.9)	74.8 (9.9)	74.6 (10.0)	74.5 (9.9)	<0.001
Higher education (%)^*^	164,294 (77.5)	90,177 (76.7)	49,743(80.4)	17,808 (76.4)	6,442 (69.7)	<0.001
Current smoker (%)	60,842 (28.7)	31,391 (26.7)	20,540 (33.2)	6,130 (26.3)	2,680 (29.0)	<0.001
Alcohol intake ≥20 g/day (%)	32,859 (15.5)	15,872 (13.5)	11,631 (18.8)	3,963 (17.0)	1,432 (15.5)	<0.001
Fatty liver (%)	56,178 (26.5)	29,628 (25.2)	18,623 (30.1)	5,944 (25.5)	2,061 (22.3)	0.003
Obesity (%)	62,114 (29.3)	30,098 (25.6)	21,345 (34.5)	7,739 (33.2)	2,856 (30.9)	<0.001
Diabetes mellitus (%)	5,724 (2.7)	2,469 (2.1)	1,917 (3.1)	909 (3.9)	471 (5.1)	<0.001
Hypertension (%)	29,043 (13.7)	13,403 (11.4)	9,960 (16.1)	4,102 (17.6)	1,719 (18.6)	<0.001
Cardiovascular disease (%)	6,996 (3.3)	3,645 (3.1)	1,856 (3.0)	979 (4.2)	462 (5.0)	<0.001
Insulin (µIU/mL)^b^	4.80 (3.27–6.92)	4.85 (3.31–6.99)	4.85 (3.29–6.97)	4.57 (3.12–6.53)	4.46 (3.00–6.50)	<0.001
Glucose (mg/dL)^a^	94.0 (14.7)	93.3 (13.9)	94.9 (15.8)	94.8 (15.1)	95.2 (15.2)	<0.001
Total cholesterol (mg/dL)^a^	194.3 (34.6)	192.6 (34.6)	196.7 (34.6)	196.0 (34.2)	195.4 (34.2)	<0.001
LDL-C (mg/dL)^a^	113.7 (30.0)	112.6 (30.1)	115.8 (29.8)	114.3 (29.3)	112.3 (29.4)	<0.001
HDL-C (mg/dL)^a^	55.6 (12.8)	55.8 (12.9)	54.5 (12.2)	56.5 (12.9)	58.6 (13.9)	<0.001
Triglycerides (mg/dL)^b^	102 (72–151)	99 (70–148)	110 (77–161)	100 (71–146)	92 (66–133)	0.895
HOMA IR^b^	1.69 (1.22–2.28)	1.69 (1.21–2.29)	1.72 (1.24–2.30)	1.67 (1.21–2.23)	1.58 (1.14–2.16)	<0.001
hs-CRP (mg/L)^b^	0.04 (0.02–0.1)	0.04 (0.01–0.09)	0.05 (0.02–0.1)	0.04 (0.02–0.09)	0.04 (0.01–0.09)	<0.001

Data are presented as ^a^mean (standard deviation), ^b^median (interquartile range), or percentage.

BMI, body mass index; BP, blood pressure; LDL-C, low-density lipoprotein cholesterol; HDL-C, high-density lipoprotein cholesterol; HOMA IR, homeostasis model assessment of insulin resistance, hs-CRP, high sensitivity C-reactive protein. Cardiovascular disease was defined as coronary heart disease or stroke.

*≥ College graduate.

**Table 3:** Risk of incident AF according to physical activity level.

PA level	Person-years	Number of events	Incident (100,000 person-years)	Age-sex adjusted HR (95% CI)	Multivariable HR (95% CI)		
					Model 1^*^	Model 2^†^	Model 3^‡^
Sedentary PA	668,949.5	129	19.3	1.00 (reference)	1.00 (reference)	1.00 (reference)	1.00 (reference)
Insufficient PA	357,963.5	107	29.9	1.05 (0.81–1.36)	1.04 (0.76–1.41)	1.01 (0.74–1.37)	1.00 (0.74–1.37)
Sufficient PA	129,670.4	49	37.8	1.26 (0.91–1.76)	1.37 (0.93–2.02)	1.32 (0.90–1.94)	1.34 (0.91–1.97)
Health-enhancing PA	48,012.9	19	39.6	1.12 (0.69–1.83)	1.27 (0.72–2.24)	1.25 (0.71–2.19)	1.27 (0.72–2.23)
P for trend				0.26	0.14	0.20	0.18

^*^Adjusted for age, sex, center, year of screening examination, smoking status, alcohol intake, and education level.

^†^Model 1 adjustments plus adjustment for BMI, diabetes, hypertension, cardiovascular disease.

^‡^Model 2 adjustments plus adjustment for hs-CRP.

PA, physical activity; AF, atrial fibrillation; hs-CRP, high sensitivity C-reactive protein; CI, confidence interval; HR, hazard ratio.

## Discussion

Our results demonstrated that PA level was not associated with AF risk. Generally, PA protects against AF by modifying traditional cardiovascular risk factors such as HTN^21^, diabetes^22^, obesity^23^, and sleep apnea, which improve cardiac structure and function^24^. Furthermore, PA has beneficial effects on age-related declines in arterial elasticity^25^. Despite these favorable risk factor modifications, several studies have suggested that young athletes who participate in endurance sports long-term or people with high-intensity PA levels have increased risk for AF^3,4,6,7^. Some studies found that AF risk is increased from 2- to 7-fold in the endurance athlete population^3,6^. However, studies of PA risk for AF have varied widely with regard to study design, adopting diverse definitions and durations of PA and different study population characteristics (such as age and sex)^26,27^. One retrospective study found a graded dose-response relationship between regular endurance PA and risk for AF in men aged >53 years^5^. However, Mozaffarian *et al*. reported a U-shaped association between PA and AF risk in adults ≥65 years^10^: moderate-intensity PA was associated with significantly lower AF incidence compared with no PA, but high-intensity PA did not significantly lower risk compared to no PA. Some authors demonstrated a J-shaped relationship between PA and the risk of AF, suggesting that AF risk was negatively associated with moderate-intensity PA, but positively associated with high-intensity PA via autonomic nervous system alterations and remodeling of the heart^28,29^. This study also found that lower resting heart rate was associated with risk of AF and could be used as an alternative measure to overcome the methodological limitations of self-reported PA^28^. Similar results in a large cohort study indicated that low resting heart rate and high-intensity PA were associated with AF^30^. Recent meta-analysis demonstrated that sedentary lifestyle significantly increases and moderate-intensity PA reduces the risk of AF in men and women^27^. A large cohort study reported that higher levels of moderate- to high-intensity leisure-time PA (for more than five hours/week) at a younger age (30 years) in men was associated with increased AF risk later in life^8^, whereas leisure time PA in older men (mean age of 60 years) did not increase the risk of AF. Frequent high-intensity PA (5–7 times/week) has been reported to increase AF risk by 20% in men <50 years old compared to those who do not engage in PA^31^. These studies suggested that long-term high-intensity PA at a younger age increases risk of AF.

Potential mechanisms for increased AF with frequent high-intensity PA include left atrial enlargement, left ventricular hypertrophy, left ventricular dilation, inflammatory changes to the atrium, and increased parasympathetic tone^32,33^. Contrary to the findings of previous studies, our study indicated that high-intensity PA was not significantly associated with increased AF risk. Despite additional analysis after reclassification of the excessive PA group among the health-enhancing group, there were no significant associations between PA and AF risk. Our study population was not at high risk for AF because of their relatively younger age and low prevalence of HTN (14%), diabetes (2.7%), and CVD (3.3%). These baseline characteristics were not significantly different from other studies^28,30^. However, direct comparisons of PA level among studies is challenging due to the diverse definitions of the intensity and duration of PA used in each study. Some studies included elite skiers^5,6^ or marathon runners^3^ over a 10-year follow-up period, and the PA level of people engaging in these sports would be higher than that garnered from leisure time PA in the general population. The average degree of self-reported PA in our study might not be as intense as that of athletes, and therefore may be insufficient to cause structural remodeling of the heart that could affect AF incidence. The beneficial effects of PA on modifiable risk AF factors, including HTN^21^, diabetes^34^, and obesity could override the potential negative effects of high-intensity PA in the general population.

Some previous studies reported gender differences in the association between PA and incident AF^27,35,36^. They found that women did not exhibit the same increased risk of AF with high-intensity PA as men. A possible explanation was that women have less pronounced atrial remodeling and lower sympathetic tone despite comparable PA compared with men^37^. Whereas AF patients were predominantly men in our study, the incidence of AF in women was too small for statistical analysis. Future large-scale studies investigating gender differences between PA and the risk of AF are needed. Another study demonstrated that height was associated with AF^9^. Although AF patients were taller compared with those without AF in our study, the relationships between height and the incidence of AF remain unclear.

Our results were in line with the Danish Diet, Cancer, and Health study, which did not demonstrate an association between working hours or PA and AF risk^38^. The study proposed that the association may be weak because publication bias has led to an overemphasis on the association between risk of AF and endurance sport activities. Some meta-analyses found no significant increase in AF with high-intensity PA^39–41^.

Our study was a general Asian population-based study with a large sample size, but a small number of AF cases. Our study had several limitations. First, PA level was self-reported. Although we used the validated IPAQ-SF, recall bias was still possible, such as over-reporting or imprecise reporting of PA. More objective and accurate measures of PA such as maximal oxygen uptake (VO_2 max_) could be used to clarify the relationship between PA and AF risk in future studies. Second, it is possible that the incidence of AF could have been underestimated; we cannot rule out the possibility that some participants classified as non-cases had paroxysmal AF. Yearly assessment of AF with only a 12-lead ECG could miss paroxysmal AF or short-duration AF in younger (<40 years) individuals. Frequent and longer ECG monitoring such as seven-day Holter monitoring, event recording, or an implantable loop recording could be more accurate diagnostic tools for paroxysmal AF. Third, there might have been selection bias because >80% of the study population consisted of employees or spouses of employees of local companies and governmental organizations, therefore limiting generalizability to the entire Korean population. Fourth, AF incidence and prevalence will likely grow as the population continues to age. There may have been too few cases of AF to validate its association with PA level because our study cohort was not at high risk for AF. Generalization of our study to other populations should be undertaken with caution. Lastly, the relatively short follow-up period could be another limitation to fully evaluating the association between PA and the risk of AF.

In conclusion, our study does not support an association between the risk of AF and PA level in a young Korean population.
