# Publisher Correction: Association between self-reported physical activity and incident atrial fibrillation in a young Korean population

**DOI:** 10.1038/s41598-019-44437-3

**Published:** 2019-06-05

**Authors:** Sung Ho Lee, Seungho Ryu, Jong-Young Lee, Dae Chul Seo, Byung Jin Kim, Ki-Chul Sung

**Affiliations:** 1 Division of Cardiology, Department of Internal Medicine, Kangbuk Samsung Hospital, Sungkyunkwan University School of Medicine, Seoul, Republic of Korea; 2 Department of Occupational and Environmental Medicine, Kangbuk Samsung Hospital, Sungkyunkwan University School of Medicine, Seoul, Republic of Korea; 3 Center for Cohort Studies, Total Healthcare Center, Kangbuk Samsung Hospital, Sungkyunkwan University School of Medicine, Seoul, Republic of Korea; 4 Department of Clinical Research Design & Evaluation, SAIHST, Sungkyunkwan University, Seoul, Republic of Korea

Correction to: *Scientific Reports* 10.1038/s41598-019-40744-x, published online 12 March 2019

The original version of this Article contained errors.

The authors Sung Ho Lee, Jong-Young Lee, Dae Chul Seo, Byung Jin Kim and Ki-Chul Sung were incorrectly affiliated with ‘Department of Occupational and Environmental Medicine, Kangbuk Samsung Hospital, Sungkyunkwan University School of Medicine, Seoul, Republic of Korea’.

Furthermore, an affiliation was omitted, which is now listed as Affiliation 1.

The correct affiliations list and affiliated authors are given below.

Affiliation 1:

Division of Cardiology, Department of Internal Medicine, Kangbuk Samsung Hospital, Sungkyunkwan University School of Medicine, Seoul, Republic of Korea

Sung Ho Lee, Jong-Young Lee, Dae Chul Seo, Byung Jin Kim & Ki-Chul Sung

Affiliation 2:

Department of Occupational and Environmental Medicine, Kangbuk Samsung Hospital, Sungkyunkwan University School of Medicine, Seoul, Republic of Korea

Seungho Ryu

Affiliation 3:

Center for Cohort Studies, Total Healthcare Center, Kangbuk Samsung Hospital, Sungkyunkwan University School of Medicine, Seoul, Republic of Korea

Seungho Ryu

Affiliation 4:

Department of Clinical Research Design & Evaluation, SAIHST, Sungkyunkwan University, Seoul, Republic of Korea

Seungho Ryu

These errors have now been corrected in the PDF and HTML versions of the Article.

